# Psychosocial adjustment in perinatally human immunodeficiency virus infected or exposed children – a Retrospective Cohort Study

**DOI:** 10.7448/IAS.19.1.20694

**Published:** 2016-06-23

**Authors:** Sarah K Zalwango, Florence N Kizza, Allan K Nkwata, Juliet N Sekandi, Robert Kakaire, Noah Kiwanuka, Christopher C Whalen, Amara E Ezeamama

**Affiliations:** 1 Directorate of Public Health and Environment, Kampala Capital City Authority, Kampala, Uganda; 2 Department of Epidemiology and Biostatistics, University of Georgia, Athens, Georgia; 3 Division of Health Protection, Office of HIV, Georgia Department of Public Health, Atlanta, Georgia; 4 School of Public Health, Makerere University College of Health Sciences, Kampala, Uganda; 5 Department of Psychiatry, College of Osteopathic Medicine, Michigan State University, East Lansing, Michigan

**Keywords:** psychosocial adjustment, depressive symptoms, distress, positive outlook, HIV

## Abstract

**Objective:**

To determine whether perinatal HIV infection and exposure adversely affected psychosocial adjustment (PA) between 6 and 18 years of life (i.e. during school-age and adolescence).

**Methods:**

We enrolled 58 perinatally HIV-infected, 56 HIV-exposed uninfected and 54 unexposed controls from Kampala, Uganda. Perinatal HIV status was determined by 18 months of age using a DNA-polymerase chain-reaction test and was confirmed via HIV rapid diagnostic test at psychosocial testing when the children were 6 to 18 years old. Five indicators of PA (depressive symptoms, distress, hopelessness, positive future orientation and esteem) were measured using validated, culturally adapted and translated instruments. Multivariable linear regression analyses estimated HIV-status-related percent differences (*β*) in PA indicators and corresponding 95% confidence intervals (CIs).

**Results:**

During school-age and adolescence, positive outlook (*β*=−3.8, 95% CI: −7.2, −0.1) and self-esteem (*β=*−4.3, 95% CI: −6.7, −1.8) scores were significantly lower, whereas depressive (*β=*11.4, 95% CI: 3.3, 19.5) and distress (*β=*12.3, 95% CI: 5.9, 18.7) symptoms were elevated for perinatally HIV-infected, compared to unexposed controls and exposed uninfected children. Similarly, positive outlook (*β=*−4.3, 95% CI: −7.3, −1.2) and self-esteem were lower for exposed controls versus HIV-unexposed children. Hopelessness was similar by perinatal HIV status. Likewise, the distress and depressive symptom levels were comparable for HIV-exposed uninfected and HIV-unexposed children.

**Conclusions:**

Perinatal HIV infection predicted higher distress and depressive symptoms, while HIV-affected status (infection/exposure) predicted low self-esteem and diminished positive outlook in the long term. However, HIV-affected status had no impact on hopelessness, suggesting that psychosocial interventions as an integral component of HIV care for infected children or primary care exposed uninfected children may improve PA and quality of life in these vulnerable groups.

## Introduction

With the greater longevity afforded by timely access to highly active antiretroviral therapy (HAART) [1], perinatal HIV infection has largely become a chronic morbidity [2] with survival into school-age, adolescence, and beyond becoming increasingly routine in most settings [3, 4]. However, the aversion of premature mortality may not translate to long-term functional survival. People living with HIV contend with HIV-specific [5, 6] and non-HIV-specific stressors throughout the life-course that lower physical health [7, 8], mental health and quality of life [9–12]. For perinatally HIV-infected (PHIV) or exposed controls, physiologic, social and psychological stress is pervasive from the gestational period [13]. Notable stressors include malnutrition, the chronic illness and premature death of relatives due to HIV, the premature assumption of responsibility for oneself and family members, contention with HIV-related stigma and discrimination, acute awareness of one's own mortality and a future tainted with above-average uncertainty [14].

The prevalence of psychiatric disorders in PHIV children from the United States ranges from 55 to 61% [15] and PHIV were four times more likely to develop psychiatric disorders relative to unexposed controls who were US children [16, 17]. A study of early versus delayed HAART in children with HIV from five low-income countries found that high levels of psychosocial distress, not HAART, predicted poor cognitive outcomes in older PHIV children [18] and suggests the importance of understanding psychosocial adjustment (PA) in HIV-affected children. Healthy PA is associated with low stress levels in children and enhances quality of life throughout the life-course [19].

The extent to which PHIV or exposed control status impairs PA in the long term in HIV-affected children from sub-Saharan Africa (SSA) is unknown. Cross-sectional investigations in SSA settings have reported a high prevalence of behavioural and emotional problems [20] and psychiatric conditions [21] in HIV-affected children but longitudinal information from the region is lacking. More information on this subject is available for HIV-affected children outside SSA but the literature is conflicted. Some studies found no HIV-related PA differences [2, 22, 23], whereas others found worse PA for PHIV versus unexposed controls [4, 24–26]. Hence, we evaluate perinatal HIV-status-related differences in five indicators of PA, which are distress, depression, hopelessness, level of positive outlook and esteem during school-age and adolescence, to inform the future need for mental health interventions in HIV-affected children. Positive outlook and high esteem are established protective factors of psychopathology, whereas high levels of distress, depression and hopelessness are known promoters of psychopathology in school-age and adolescence [27]. We hypothesize that both PHIV and exposed controls will experience poorer PA than unexposed controls during school-age and adolescence.

## Methods

### Study design, population, inclusion/exclusion criteria

A retrospective cohort study was implemented including 168 children with and without perinatal HIV infection/exposure enrolled from a Community Clinic in Kampala Uganda between March 20, 2014, and July 30, 2014. We specifically targeted school-aged children (i.e. those 6–18 years old) for inclusion in this study. Child and caregiver eligibility criteria included (1) documented delivery of the index child in a hospital setting within Kampala or its nearby rural between 1996 and 2008, (2) determinable HIV status of birth mother and index child during the index pregnancy, delivery or breastfeeding of the index child using objective medical records, (3) willingness to undergo testing to verify current HIV sero-status (if HIV negative) and (4) provision of parental consent and child assent for study participation. Eligible children were initially enrolled on a first-come, first-served basis using a convenience sampling method. We combined this primary approach with snow-ball sampling by inviting and enrolling the multiple eligible children of a given caregiver. Children for whom official birth records were unavailable and whose perinatal HIV status could not be objectively determined were excluded.

### Statement of ethical clearance

Ethical approval for the implementation of this study was provided by the institutional review boards of the University of Georgia (IRB Protocol # 0196), the Makerere University School of Public Health (IRB Protocol # 010). Further regulatory approval was obtained from the Uganda National Council for Science and Technology (Protocol # HS 1613). All caregivers provided written informed consent and children provided assent for study participation.

### Measurement of psychosocial adjustment indicators

Prior to the main study, structured questionnaires for each indicator were translated and phrases culturally adapted as appropriate to the study setting. First, the instruments were translated from English to Luganda by a team of bilingual doctoral or masters students, each working independently. Then, two other students who were not familiar with the source documents were asked to translate the instruments from Luganda back into English. At each stage of this process, discussions were conducted by the principal investigator, translators and investigators in the field to ensure that the instruments were culturally valid and matched the intent of the original instruments. The instruments were then used in a pilot assessment on 15 children (five PHIV, six exposed controls and four unexposed controls) aged 6 to 18 years old. Instruments were administered twice to each child 14 to 21 days apart by two research assistants who alternated between visits. Psychometric properties (internal consistency among individual questions, inter-rater and test-retest reliabilities) were calculated to measure the adequacy of the respective PA assessment in this setting using the %INTRACC macro [28]. Final questionnaire instruments were modified in light of pilot activities, where appropriate, to ensure the use of linguistically and culturally relevant phrases. The final questionnaire was administered once to an additional sample of 153 children. Each indicator is a summed composite per developer instructions and each is analyzed as a linear variable with increasing values denoting larger quantities of the respective psychosocial measures [29].

Depressive symptom score was defined as the summed composite of six questions using the modified depression scale (MDS) to measure the frequency of depressive symptoms over the past month [30]. Symptoms included sadness, irritable, grouchy or bad mood, hopelessness about the future, impaired sleep and difficulty concentrating, and each was scored on a scale of never (1), seldom (2), sometimes (3), often (4) and always (5). A distress score was derived as the sum of the responses to 12 questions per the Distress Weinberger Adjustment Inventory [31]. Hopelessness is measured as negative future expectations using the Children's Hopelessness Scale as a sum of 17 individual questions to which a “no” (score=1) response represents hopelessness and a “yes” (score=0) response indicates lack of hopelessness. Positive outlook measured each child's future aspiration as the sum of six questionnaire items included in the Positive Outlook-Individual Protective Factors index. Global and area-specific esteem is evaluated via ten questions reflecting a child's assessment of their worth and importance among peers (*n=*3), as students (*n=*3), and as family members (*n=*4) per the Hare Area-specific self-esteem scale. Respective instruments have been developed and validated in children and adolescents from largely non-SSA settings. Extensive details about each scale including the approach to scoring is described elsewhere [29].

### Perinatal HIV status

Perinatal HIV exposure/infection status was determined by DNA-polymerase chain reaction by 18th month of life; HIV rapid diagnostic test confirmed status in all HIV-negative children during psychosocial assessment. Perinatal HIV is defined in three categories as PHIV, exposed controls and unexposed controls.

### Other measures

Laboratory investigations on children included complete blood counts, assessments for helminthes, intestinal parasites and malaria infections. Medical officers documented comorbid conditions at enrolment as part of study health assessment. Medical chart review was implemented to determine birth weight and Apgar score and verify maternal HIV status in pregnancy. The following confounders were measured as child health and demographic factors (sex, age, current bed-net use, birth weight, Apgar score, child nutrition, infection and hematologic status indicators) caregiver's health, demographic and behavioural factors (sex, age and education status, wealth score, BMI, self-reported health, social support score, anxiety score, depressive symptoms score and alcohol use) were measured at enrolment using structured questionnaires.

### Statistical analyses

We compared mean PA scores by perinatal HIV status and implemented bivariate analyses to identify non-HIV factors crudely associated with PA. For descriptive analyses only, the analysis of a variance (ANOVA) test of no difference in average PA scores by perinatal HIV status was implemented using *F*-tests for normally distributed linear variables. If the normality assumption was violated, the Kruskal-Wallis test of differences in median by HIV status was implemented. Similarly, the socio-demographic characteristics of PHIV, exposed controls and unexposed controls were summarized by chi-square tests for the categorical variables. We implemented multivariable linear regression models and estimated the differences (β) in PA scores as well as associated 95% confidence intervals (CIs), using a generalized estimated equations (GEE) approach with an identity link. These GEE-based models provide unbiased estimates of mean differences that are efficiently estimated per asymptotic theory by maximizing the normal log-likelihood [32]. Empirical standard errors were used to calculate robust CIs for all estimated βs. We clustered analyses at the household level and specified an exchangeable working correlation matrix to control for lack of independence of children of the same caregiver.

For multivariable modeling, we considered factors associated with PA at a *p*-value of ≤0.20 from descriptive analyses as potential confounders in multivariable analyses. For all multivariable analyses, missing confounder values were addressed using the missing indicator method so that the analytic sample included all children with data on perinatal HIV status and PA. All analyses were implemented in SAS version 9.4. Our presented results include unadjusted estimates and results from a pair of nested multivariable models. Multivariable models controlled for child and caregiver socio-demographic, behavioural and health factors (multivariable model 1) and additional control for caregiver health and caregiver psychosocial factors, social support and perceived social standing (multivariable model 2).

## Results

Among the 15 children included in the instrument refinement pilot-study, the set of questions used for measuring child distress, hopelessness, school esteem and global esteem demonstrated good internal consistency, whereas those for the assessment of depressive symptoms, peer esteem and home esteem demonstrated acceptable consistency in the assessment of respective PA constructs. The consistency between raters in the administration of the respective questionnaires was fair to moderate for global- and area-specific esteem, good for distress assessment, and fair for all other scales with the exception of assessments for hopelessness that demonstrated poor level of agreement between the raters. Over 14 to 21 days of repeated testing, the stability of child PA rating was excellent for global distress scale assessment, and fair to good for the depressive symptom score, home/family esteem, school esteem and global self-esteem scales. It was unacceptable or poor for positive outlook, hopelessness and peer-esteem scales (Table 1).

**Table 1:** Psychometric properties of the psychosocial adjustment status questionnaires among 15 children in the pre-pilot phase

	Inter-rater reliability^a^	Test-retest reliability^b^	Internal consistency^c^
	ICC	ICC	Cronbach's α
Depressive symptom score	0.37	0.48	0.63
Distress score	0.62	0.76	0.72
Hopelessness	0.13	0.22	0.81
Positive outlook	0.30	<0.2	0.53
Area-specific self-esteem			
Peer esteem	0.21	0.35	0.68
Home/family esteem	0.30	0.51	0.68
School esteem	0.45	0.63	0.77
Global self-esteem	0.32	0.53	0.82

ICC=intra class correlation.

a ICC≥0.81 (very good); 0.61≤ICC<0.8 (good); 0.41≤ICC<0.6 (moderate); 0.21≤ICC<0.4 (fair); ICC<0.2 (poor).

b ICC>0.75 (excellent); 0.40≤ICC≤0.75 (fair to good); ICC<0.4 (poor).

c α≥0.9 (excellent); 0.7≤α<0.9 (good); 0.6≤ α<0.7 (acceptable); 0.5≤α<0.6 (poor); α<0.5 (unacceptable).

Of the 168 children enrolled, three were excluded for lack of measurement of any PA indicator. Analytic sample size, including children with HIV and respective adjustment indicators, ranged from 165 to 160 depending on the PA indicator. Children's ages, sex, birth weights, Apgar scores at birth, short-term nutritional status and coincident infections at enrolment into the study were similar by perinatal HIV status. Likewise, caregiver sex, age, alcohol use, perceived social standing, social support, anxiety and depressive symptom scores were similar by child HIV status. However, height-for-age was significantly lower, whereas anaemia was more prevalent in PHIV children. The frequency of children's current bed-net use was highest in unexposed controls, followed by PHIV and lowest in exposed control children. Caregivers of HIV-affected children (PHIV and exposed controls) had least average material wealth cores and were more likely to have primary or lower education (Table 2).

**Table 2:** The health, behavioural and socio-demographic description of the study sample overall and by perinatal HIV status in 6- to 18-year-old children from Kampala, Uganda

	Overall 165 (100)	HIV-unexposed control 53 (32.1)	HIV-exposed control 54 (33.3)	HIV-infected 58 (34.5)	*p*^a^
**Child health and demographic factors**					
Female child	77 (45.8)	28 (51.9)	23 (41.1)	26 (44.8)	0.52
Child current bed-net use	125 (76.7)	47 (88.7)	34 (60.7)	44 (81.5)	<0.01
Age, years					
Mean (SD)	10.8 (3.49)	10.61 (3.82)	10.57 (3.67)	11.2 (3.0)	0.27
<7	31 (18.45)	12 (22.2)	12 (21.4)	6 (10.5)	0.20
7–9	49 (29.2)	17 (31.5)	18 (32.1)	14 (24.6)	
10–12	40 (23.8)	9 (16.7)	10 (17.9)	21 (36.8)	
13–18	48 (28.6)	16 (29.6)	16 (28.6)	16 (28.1)	
Child schooling status					
Not in school	7 (4.2)	0 (0)	3 (6.0)	4 (7.0)	0.15
In school	158 (95.8)	53 (100)	51 (94)	54 (93.0)	
Relationship with caregiver					
Mother	117 (70.5)	39 (72.2)	45 (80.4)	33 (58.9)	0.02
Father	16 (9.6)	04 (7.4)	7 (12.5)	5 (8.9)	
Other relative	33 (19.9)	11 (20.4)	4 (7.1)	18 (32.2)	
Birth weight (kg), mean (SD)	3.41 (0.60)	3.39 (0.62)	3.44 (0.59)	3.40 (0.59)	0.95
Apgar score <10, *n*, %	43 (29.9)	12 (25.5)	17 (32.1)	14 (31.8)	0.73
Child nutrition					
HAZ, mean (SD)	−0.81 (1.74)	−0.59 (1.70)	−0.35 (1.70)	−1.43 (1.66)	0.01
BMIZ, mean (SD)	−0.97 (1.58)	−0.72 (1.14)	−1.30 (1.70)	−0.88 (1.78)	0.67
Infection					
% Malaria/helminth infection	12 (7.1)	4 (7.4)	5 (8.9)	3 (5.2)	0.74
% Intestinal protozoa	15 (8.9)	4 (7.4)	4 (7.4)	7 (12.1)	0.68
Hematologic status indicators					
Hemoglobin (g/dl), mean (SD)	12.8 (1.8)	12.9 (1.2)	13.4 (2.3)	12.3 (1.6)	0.10
Microcytic/macrocytic anaemia,^b^ *n* (%)	73 (47.7)	27 (51.9)	17 (31.4)	29 (60.9)	0.01
**Caregiver's health and demographic factors**					
Educational status					
<Primary education	66 (40.5)	9 (17.0)	30 (54.6)	27 (49.1)	<0.01
Primary education	30 (18.4)	11 (20.8)	9 (16.4)	10 (18.2)	
Any O level or higher	67 (41.1)	33 (62.2)	16 (29.0)	18 (32.7)	
Female caregiver, *n* (%)	138 (82.1)	44 (81.5)	45 (80.4)	49 (84.5)	0.84
Alcohol use	8 (4.8)	2 (3.7)	3 (5.4)	3 (5.2)	0.91
Age (years), mean (SD)	39.1 (10.3)	38.3 (8.6)	39.6 (8.3)	39.3 (13.3)	0.63
Wealth score, mean (SD)	2.7 (2.1)	4.2 (2.0)	1.9 (1.8)	2.1 (1.8)	<0.01
BMI (kg/m^2^), mean (SD)	24.3 (4.3)	25.9 (4.5)	22.8 (3.2)	24.4 (4.6)	0.07
Low caregiver self-reported health^c^	91 (54.2)	23 (42.6)	32 (57.1)	36 (62.1)	0.10
Perceived social standing, mean (SD)	3.3 (2.0)	3.7 (2.1)	3.0 (1.9)	3.3 (1.9)	0.22
Social support score, mean (SD)	22.9 (6.3)	23.2 (5.8)	24.0 (6.3)	21.5 (6.6)	0.15
Anxiety score, mean (SD)	20.7 (7.8)	21.4 (7.5)	19.5 (8.2)	21.3 (7.7)	0.94
Depressive symptoms score, mean (SD)	29.2 (9.9)	29.5 (11.1)	28.9 (9.8)	29.2 (9.0)	0.88

a *p* Values for difference in means are derived from analysis of variance across HIV groups. *p* Values for difference in proportion across HIV groups are derived from chi-square tests.

b Macrocytic anemia is linked to micronutrient deficiencies e.g. B-12 deficiency (*n=*22), microcytic anemia indicates iron deficiency anemia (*n=*65). Some children had both types of anemia concurrently.

c Based on caregiver subjective rating of own health as fair/poor versus good, very good or excellent.

Among PHIV children, the median age at first CD4 assessment in the Kawaala health center was four years (range: 0.1–13.7) with at a median CD4 cell count of 342 (range: 6–2013 cells/µL) at first assessment. At the time of enrolment, the median time in care at the health center was 5.9 (range: 0.6–16.6) years and 77.6% of the sample were on highly active anti-retroviral therapy. Absolute CD4 is usually assessed bi-annually per SOC with the most recent measurement (median=772, range: 63–2521 cells/µL) obtained within 3.4 (range: 0–32) months of enrolment into the study. (Data not shown.)

**Figure 1:**
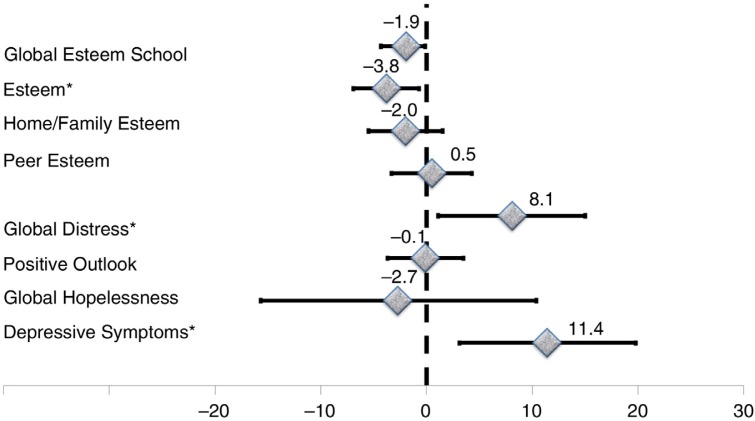
Differences in psychosocial adjustment indicators for school-age children and adolescents for perinatally HIV-infected compared to HIV-exposed control children from Kampala, Uganda. Asterisks identify indicators that are significantly different across groups.

**Figure 2:**
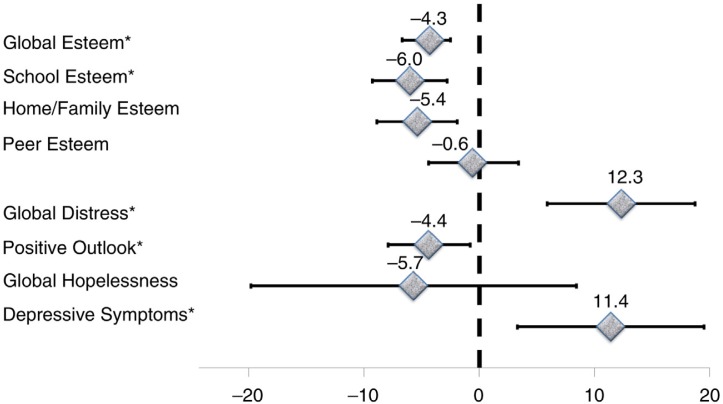
Differences in psychosocial adjustment indicators for school-age children and adolescents for perinatally HIV-infected children relative to HIV-unexposed control children from Kampala Uganda. Asterisks identify indicators that are significantly different across groups.

**Figure 3:**
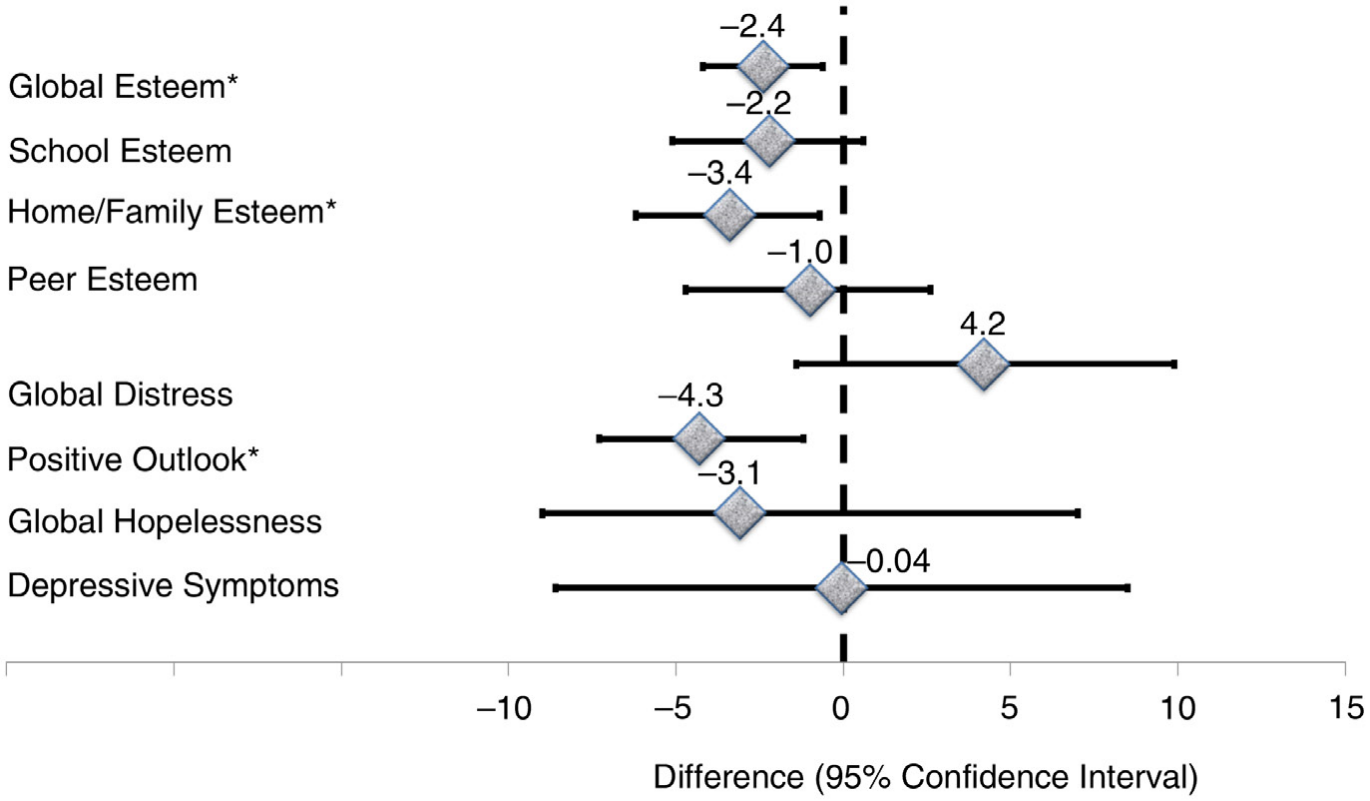
Differences in psychosocial adjustment outcomes for school-age children and adolescents for perinatally HIV-exposed negative compared to HIV-unexposed children from Kampala, Uganda. Asterisks identify indicators that are significantly different across groups.

For the entire sample, a descriptive unadjusted summary of median scores by perinatal HIV status is presented in Table 3. Median depressive symptoms and distress scores were higher for PHIV, whereas the average positive outlook and esteem scores were lower in PHIV versus other groups. Scores in the respective PA measures were similar for exposed controls and unexposed controls. There were no differences between the three groups with respect to hopelessness and peer-esteem scores (Table 3). Perinatal HIV-related differences in respective PA indicators are shown in Table 4 with three explicit comparisons: exposed controls versus PHIV (Figure 1), unexposed controls versus PHIV (Figure 2) and unexposed controls versus exposed controls (Figure 3). The average depressive symptom and global distress scores were, respectively, 11.4 and 12.3% elevated, whereas the mean scores on positive outlook, home/family esteem, school esteem and global esteem were lower by an average of 4.4, 5.4, 6.0 and 4.3% for PHIV, relative to unexposed controls children. However, global hopelessness scores and peer esteem was statistically equivalent for PHIV and unexposed controls. In comparison to exposed controls, PHIV children average school esteem scores was significantly lower (−3.8%) and both depressive symptom and distress scores were significantly elevated by an estimated 11.4 and 8.1%, respectively, for PHIV compared to exposed controls children. There was no difference between PHIV and exposed controls with respect to average adjustment levels in the areas of hopelessness, positive outlook, peer esteem, family/home esteem and global esteem. Exposed control children exhibited similar levels of depressive symptoms and had comparable levels of hopelessness, peer and school esteem scores as unexposed controls children. However, their distress scores were 4.2% elevated (but not statistically significant), while their global and family/home esteem scores as well as positive outlook scores were significantly lower in comparison to unexposed controls. All associations were robust to adjustment for multiple child and caregiver factors, including nutritional status, educational status and caregiver depression, anxiety and social support (Table 4, Figures 1–3).

**Table 3:** Basic description of psychosocial adjustment indicators by perinatal HIV status among 6- to 18-year-old Children from Kampala, Uganda

	Overall *n* (%) 165 (100)	HIV-unexposed control *n* (%) 53 (31.9)	HIV-exposed control *n* (%) 54 (33.1)	HIV-infected *n* (%) 58 (34.9)	
					
	Median (IQR)	Median (IQR)	Median (IQR)	Median (IQR)	*p*^a^
Depressive symptom score (*n*=161)	9 (5)	8 (11)	8 (4)	10 (6)	0.01
Distress score (*n*=162)	18 (11)	17 (11)	16.5 (10)	20 (13)	0.03
Hopelessness (*n*=165)	1 (2)	1 (1)	1 (2)	1 (4)	0.71
Positive outlook (*n*=163)	19 (3)	20 (3)	19 (3)	19 (3)	0.02
**Area-specific self-esteem**					
Peer esteem (*n*=164)	8 (1)	8 (1)	78 (1)	8 (1)	0.30
Home/family esteem (*n=*164)	12 (1)	12 (2)	12 (1)	12 (1)	0.20
School esteem (*n*=160)	9 (0)	9 (1)	9 (0)	9 (0)	<0.01
Global esteem (*n*=164)	29 (2)	29 (3)	29 (2)	29 (1)	0.02

IQR=inter-quartile range.

a *p* Values are derived for null hypothesis of no HIV-related differences in respective psychosocial adjustment indicators. Given skewed distribution, differences in median are estimated using non-parametric test for respective psychosocial across the three HIV groups using the Kruskal-Wallis test.

**Table 4:** Perinatal HIV status in relation to psychosocial adjustment outcomes among 6- to 18-year-old children from Uganda^a^

	HIV-exposed control versus HIV-unexposed control children			HIV-infected versus HIV-unexposed control children			HIV-infected versus HIV-exposed control children			
				
	Crude association	Multivariate model 1^b^	Multivariate model 2^c^	Crude association	Multivariate model 1^b^	Multivariate model 2^c^	Crude association	Multivariate model 1^b^	Multivariate model 2^c^	
										
	Difference (95% CI)	Difference (95% CI)	Difference (95% CI)	Difference (95% CI)	Difference (95% CI)	Difference (95% CI)	Difference (95% CI)	Difference (95% CI)	Difference (95% CI)	*R*^2^
Depressive symptoms	0.1 (−6.2, 6.5)	1.1 (−7.7, 9.9)	−0.04 (−8.6, 8.5)	9.6 (3.0, 16.2)	11.4 (2.8, 20.0)	**11.4 (3.3, 19.5)**	9.5 (2.0, 16.9)	10.3 (2.2, 18.5)	**11.4 (3.1, 19.8)**	0.10
Global hopelessness	−1.6 (−15.6, 12.4)	−2.9 (−18.2, 12.3)	−3.1 (−19.6, 13.5)	−0.7 (−13.8, 12.4)	−5.9 (−20.1, 8.3)	−5.7 (−19.8, 8.4)	0.9 (−10.4, 12.1)	−3.0 (−14.6, 8.6)	−2.7 (−15.7, 10.4)	0.08
Positive outlook	−2.8 (−6.3, 0.8)	−1.7 (−4.4, 1.0)	−**4.3 (**−**7.3**, −**1.2)**	−3.8 (−7.2, −0.1)	−3.5 (−7.0, −0.01)	−**4.4 (**−**7.9**, −**0.8)**	−0.9 (−4.2, 2.4)	−1.8 (−5.3, 1.7)	−0.1 (−3.7, 3.5)	0.13
Global distress	0.8 (−4.3, 5.9)	0.9 (−4.7, 6.4)	4.2 (−1.4, 9.9)	8.1 (1.6, 14.6)	10.5 (3.6, 17.5)	**12.3 (5.9, 18.7)**	7.3 (1.1, 13.5)	9.7 (3.1, 16.2)	**8.1 (1.1, 15.0)**	0.20
Area-specific self-esteem										
Peer esteem	−1.6 (−4.7, 1.6)	−1.4 (−4.8, 2.1)	−1.0 (−4.7, 2.6)	−1.8 (−4.8, 1.3)	−1.0 (−4.7, 2.8)	−0.6 (−4.4, 3.4)	−0.2 (−3.3, 3.0)	0.4 (−3.6, 4.4)	0.5 (−3.3, 4.3)	0.04
Home/family	−1.5 (−4.9, 1.8)	−1.7 (−4.8, 1.5)	−**3.4 (**−**6.2**, −**0.7)**	−3.2 (−6.3, −0.1)	−4.8 (−8.5, −1.1)	−**5.4 (**−**8.9**, −**1.9)**	−1.7 (−5.0, 1.7)	−3.1 (−6.9, 0.6)	−2.0 (−5.5, 1.5)	0.20
School esteem	−1.2 (−4.4, 2.0)	−1.6 (−4.8, 1.6)	−2.2 (−5.1, 0.6)	−4.5 (−7.7, −1.4)	−6.1 (−9.6, −2.6)	−**6.0 (**−**9.3**, −**2.8)**	−3.4 (−6.3, −0.4)	−4.5 (−7.8, −1.2)	−**3.8 (**−**6.9**, −**0.7)**	0.21
Global esteem	−1.5 (−4.0, 1.0)	−1.6 (−3.9, 0.7)	−**2.4 (**−**4.2**, −**0.5)**	−3.2 (−5.5, −0.9)	−4.1 (−6.8, −1.4)	−**4.3 (**−**6.7**, −**1.8)**	−1.7 (−4.0, 0.6)	−2.5 (−5.2, 0.1)	−1.9 (−4.3, 0.5)	0.20

a All estimates are derived from a GEE linear regression model with Psychosocial adjustment factor as a linear outcome variable. Analysis was clustered at the household level and unstructured covariance matrix was assumed to account for non-independence of children from the same households.

b The base multivariate model 1 is adjusted for: caregiver socio-demographic (age, sex and education) and child-socio-demographic and behavioural health factors (age, sex, anemic versus non-anemic status, and current versus non-current bed-net use).

c Multivariate model 2 is further adjusted for caregiver BMI, social support and perceived social standing in addition to the factors adjusted for in model 1.

## Discussion

We investigated perinatal HIV exposure/infection as a predictor of differences in five indicators of PA, which are depression, distress, hopelessness, positive future orientation and esteem, in Ugandan children between 6 and 18 years of age. Consistent with our study hypothesis, we found that HIV-affected (meaning exposed control and PHIV) children have significantly lower self-esteem and were substantially less optimistic about the future than unexposed control children. However, HIV-affected children were no different from unexposed controls with respect to hopelessness. Contrary to our expectations, HIV-affected children exhibited insignificantly lower average levels of hopelessness than unexposed control children during the school-age years. This finding is suggestive of the existing resilience of HIV-affected children in the face of significant deficits in four of the PA indicators, compared to unexposed controls. This finding is similar to previous reports of the presence of higher emotional and behavioural problems, and yet greater coping ability, for Ugandan school-age orphans compared to non-orphans [33]. Thus, HIV-affected children in this SSA context, despite their psychosocial disadvantage, maybe growing up in a background culturally and socially rich in resilience factors that could be enhanced with specific interventions to improve their long-term functional capacity.

The role of hope and a positive outlook, as correlates of long-term resilience with the potential to enhance functional survival in an era of long-term survival with HIV, has been poorly explored. As is increasingly recognized in psychotherapy [34], the presence of hope and positive future aspirations may be a crucial common denominator in the long-term achievement or functionality among survivors of all kinds, including HIV-affected children [35]. There are no comparative data on this subject to contextualize our findings in HIV-affected children, to the best of our knowledge. However, one study among youth at high risk of HIV infection within the US justice system provides proof of the principle that interventions grounded in the theory of planned behaviour modifications are feasible, and such interventions differentially benefit adolescents who report a lower positive outlook at enrolment [36]. Hence, the data from our study are encouraging in the sense that HIV-affected and unaffected children in the study setting were comparable in terms of hopefulness and hopelessness. Specific interventions to enhance a positive future outlook may therefore be necessary to realize the salutary potential inherent in the relatively lower levels of hopelessness HIV-affected children.

In line with our hypothesis, we provide empirical evidence of significantly higher depressive symptoms and distress in PHIV compared to HIV-uninfected African children. Specific comparative data on this subject is lacking for populations from SSA, although recent studies from the region in PHIV only note that psychosocial challenges were highly prevalent among PHIV and therefore warrant specific evaluation and intervention [21, 37, 38]. A recent review on the mental health of youth living with HIV is informed by data, largely from HIV cohorts in the United States [39]. Even in these non-SSA settings, implemented studies varied substantially in terms of methodology, PA measures, instruments used and perinatal HIV groups. Our finding for distress and depression is in line with previous reports of PHIV-related higher scores on depressive symptoms and anxiety among school-aged American children using a different battery of tests [40]. However, contrary to our results, unexposed controls have not been consistently associated with lower PA in the majority of studies implemented in the United States and elsewhere [39]. Specifically, higher rates of impaired psychological adjustment in unexposed controls compared to PHIV children from the United States have been reported [23], and the data from the pediatric HIV/AIDS cohort study (PHACS) have largely found no differences or superior mental health status for PHIV compared to exposed controls [41–43].

Systematic investigations of PA HIV-affected children in school-age or beyond are lacking, particularly for SSA. As for other chronic conditions, a high prevalence of low esteem, depression, distress, negative future orientations and hopelessness may be expected, given PHIV [44]. We extend existing literature by providing the first comparative study on this subject in HIV-affected relative to HIV-unaffected children from SSA. As a whole, our data suggests that HIV-affected children are similar to PHU in terms of hopefulness. Exposed controls are no different from unexposed controls with respect to depression and distress levels, but like PHIV, significantly lower levels of positive outlook and self-esteem are observed in school-aged children and adolescents. PHIV in particular, live with elevated levels of depressive symptoms and distress, more than exposed controls and unexposed controls. Thus, we confirm previous reports in children with PHIV that high levels of psychosocial distress, and not HAART, predicted sub-optimal development [18]. Hence, the long-term benefit of HAART in PHIV may be moderated by psychosocial distress, high levels of depressive symptoms, low esteem and low levels of positive outlook.

The strengths of this investigation include the use of a retrospective cohort design permitting inference regarding the temporal sequence between HIV status and PA indicators, rigorous control for multiple potential confounders and the use of instruments of known psychometric properties in the study setting. However, the following limitations should lead to cautious interpretation of our data. There is an inability to establish that observed HIV-related differences are clinically significant as well as indications of the need for further refinement/adaptation of some of the PA tools despite acceptable or higher levels of internal consistency of included questions. Specifically, our instrument refinement study suggests the possibility of rater-dependent differences in the administration of the hopelessness questionnaire instrument and unacceptable stability in the responses of children over the inter-test period for hopelessness and positive outlook questionnaires. We note that the realized low test-retest and inter-rater reliabilities of some measures may have partly reflected our enrolment approach into this study. Initial psychosocial testing was implemented on the day that children with active morbidity, that required clinical consultation, were presented at pediatric wards. Repeat testing was scheduled and implemented two to three weeks later, when most children were in relatively good health. Thus, the low reliability scores in some instruments (hopelessness and positive outlook) may partly reflect differences in acute morbidity for the index child at initial and subsequent testing. Additional limitations include small sample size, which might have impacted the statistical power of this study and the clinic-based enrolment of control groups. The latter might have led to the recruitment of materially less healthy control groups, rather the inclusion of true community control groups or healthier HIV-infected children connected to care. The potentially poor representativeness of our sample could result in the over- or under-estimation of differences between HIV groups. Future investigations on this subject of HIV-affected and unaffected children will benefit from the inclusion of non-clinic-based controls and the robust validation of all instruments and repeated rater training, as necessary, particularly in the tools for the assessment of future orientations and hopelessness.

In summary, we provide comparative evidence of the perinatal HIV status associated with differences in PA among SSA school-age children and adolescents. Our findings highlight the vulnerability of HIV-affected children from SSA to lower PA in comparison to their HIV-unaffected peers. Larger longitudinal studies are needed to evaluate the clinical significance of the HIV-related differences in PA observed. Data from our study suggests that psychosocial interventions, as a complimentary component of HIV care in HIV-infected or HIV-affected children, may enhance the quality of HAART-associated longevity gain or functional survival in these vulnerable populations in the long term.
